# The development of working life expectancy without musculoskeletal diseases against the backdrop of extended working lives

**DOI:** 10.1038/s41598-024-58650-2

**Published:** 2024-04-04

**Authors:** Juliane Tetzlaff, Jelena Epping, Jona Theodor Stahmeyer, Falk Liebers, Janice Hegewald, Stefanie Sperlich, Johannes Beller, Fabian Tetzlaff

**Affiliations:** 1 Medical Sociology Unit, Hannover Medical School, Carl-Neuberg Str. 1, 30625 Hanover, Germany; 2 AOK Niedersachsen- Statutory Health Insurance of Lower Saxony, Hannover, Germany; 3 Federal Institute for Occupational Safety and Health (BAuA), Berlin, Germany; 4 Division of Social Determinants of Health, Department of Epidemiology and Health Monitoring, Robert Koch-Institute, Berlin, Germany

**Keywords:** Diseases, Occupational health, Public health, Epidemiology

## Abstract

Musculoskeletal diseases (MSDs) are a major predictor of early retirement. Against the backdrop of the extension of working life, we investigated time trends and educational inequalities in years spent in the labour market free of MSD. Based on German statutory health insurance data (N = 3,405,673), total life years free of MSD (Healthy Life Expectancy, HLE) and years spent in the labour force free of MSD (Healthy Working Life Expectancy, HWLE) were estimated for three periods (2006–2008, 2011–2013, 2016–2018) using multistate analyses. Educational inequalities (8 to 11 vs. 12 or more years of schooling) are reported for 2011–2013. HLE decreased slightly over time in all genders. HWLE in women increased, while it remained rather constant in men. Over time, the share of years in the labour force spent free of MSD declined continuously. People with lower education had lower HLE and HWLE than individuals with higher education. With respect to musculoskeletal diseases, the increase in disease-free working life years cannot keep pace with the extension of working life, resulting in an increasing proportion of years spent in impaired musculoskeletal health in the labour market. Effective prevention strategies are needed, focusing especially on individuals with lower educational attainment.

## Introduction

As in many high-income countries, the German population is aging rapidly. To reduce the growing burden on the pension system, many governments have raised the statutory retirement age. In Germany, the current statutory retirement age is 66 for the birth cohort 1958 and is set to rise up to age 67 until 2031. As a result, the Working Life Expectancy (WLE, i.e. the expected number of years spent economically active) in Germany increased steadily during the last two decades with increases being stronger in women^1–4^. This development raises the question of whether the health of the older population allows for prolonged employment.

When assessing the possibilities and limits of prolonging working lives from a health perspective, the question of changes in the healthy working lifespan over time is crucial: This research can lead to important insights into whether the years of good health in the labour market increase or whether it is mainly the years of poor health that increase as working life is extended. To study this, Healthy Working Life Expectancy (HWLE) has been introduced as an indicator to depict the number of expected life years spent in the labour market in good health^5^.

Musculoskeletal diseases (MSD) are one of the major contributors to disability and pain worldwide^6–8^. The international literature clearly shows that MSD are associated with reduced work ability, an increased risk of disability pension, unemployment, or similar paths to early exit from working life^9–11^. In Germany, MSD are also a major cause of sick leave^12^, which highlights the importance of considering HWLE defined as years spent free of MSD. However, studies on HWLE with respect to specific single diseases that often cause early labour market exists, including musculoskeletal diseases, are lacking so far. Our study aims to narrow this gap by analysing time trends and educational inequalities in Healthy Working Life Expectancy in terms of life years spent in the labour market free of MSD. In order to gain insights into the general development of musculoskeletal health in the population, temporal trends and educational inequalities in the MSD-free life span are also investigated.

Overall, research on HWLE is still very limited^13^. Focusing on long-standing illnesses, mental health, disability, self-rated health (SRH) or health-related quality of life (HRQoL), studies reported increases in HWLE^3,14^, though years spent in labour in poor health increased as well^3,14, 15^. So far, only one study reported trends in HWLE based on specific chronic diseases^16^. Boissonneault and Rios found that working life years are increasingly spent with chronic diseases in many countries^16^. For Germany, research on trends in HWLE is even more limited. So far, there are only two studies that found increases in HWLE based on SRH and HRQoL. Here too, this increase is accompanied by an increase in working life years in poor health^3,17^. Furthermore, it was shown that considerable social inequalities in HWLE exist to the disadvantage of persons with lower educational attainments and lower occupational position, which are most pronounced at older age^4,17, 18^.

When analysing trends in HWLE or healthy life years, focusing on specific diseases can add to the current state of research in several ways. If general health indicators are used, such as disabilities, SRH, etc., the underlying diseases are unknown. This makes it difficult to implement targeted prevention measures that are tailored to the specific needs of the affected population. Analysing HWLE based on the absence of specific diseases can help to identify specific prevention needs and to quantify the impact of the respective diseases on the healthy working lifespan. Against this background, the study aims to contribute to the current research by addressing the following research questions:

- How did life expectancy free of musculoskeletal diseases (MSD-free LE) develop over time in men and women?
- How did musculoskeletal-disease free WLE (MSD-free WLE) develop over time in men and women?
- What educational inequalities exist in MSD-free LE and MSD-free WLE with respect to musculoskeletal diseases?

## Methods

### Data

We used the data of a large German statutory health insurance provider that includes information on ICD-10 diagnoses, mortality, age, labour force status, and education of 3,405,673 individuals living in the federal state Lower Saxony between 2006 and 2018. The statutory health insurance represents an essential pillar of the German social security system. Below a certain income threshold, statutory health insurance is mandatory to all, while individuals with a higher income above ~ 65′000 EUR may choose a private health insurer. Because this income threshold is quite high, about 90% of all inhabitants are insured within the statutory system^19^. Statutory health insurance covers all medical treatments, medications, and procedures essential to health. The fees are set to 14.6% of the individual income but were hold constant in absolute terms above a certain income threshold (59.850 € in 2023)^20^. The data are comparable to the total German population in terms of age and gender distributions. However, since private insurance is usually only accessible with higher incomes, individuals with higher socioeconomic status are underrepresented^35^. The data contain individual insurance histories, which makes it possible to identify the exact date of events, e.g. when an individual changes the labour force status, drops out from the insurance, or dies.

Our study is based on claims data, i.e. on routinely collected data of a statutory health insurance provider. The federal law regulates the use of this data for scientific purposes. All data was fully anonymised before we accessed them. Therefore, no ethical approval or consent to participate is required. We confirm that all methods were carried out in accordance with the relevant guidelines.

### MSD incidence and recovery

We used inpatient and outpatient ICD-10 diagnoses (M00–M99) to identify individuals diagnosed with MSD. Individuals were considered as incident if a diagnosis of at least one of the subgroups of MSD (arthropathies (M00–M25), systemic connective tissue disorders (M30–M36), dorsopathies (M40–M54), soft tissue disorders (M60–M79), osteopathies and chondropathies (M80–94), other disorders of the musculoskeletal system and connective tissue (M95–M99)) was confirmed by a second diagnosis code in another quarter within the same year. This is considered as standard to produce robust estimates from health insurance claims data^21^. Furthermore, incidence was only coded if the diagnosis was preceded by a diagnosis-free look-back period of at least 1 year in order to distinguish between incident and prevalent MSD cases. This approach was evaluated in a previous methodological study^22^ and applied in several other studies (e.g.^23,24^), suggesting that time trends and social inequalities in incidence can be well depicted based on German statutory health insurance data. Since some of the most prevalent MSD are not chronic progressive and allow for full recovery (e.g. back pain (ICD-10 diagnosis M54: with 21% in men and 18% in women the most frequent diagnosis among MSD-prevalent persons), muscle pain (covered for example by M79: with 2% in men and 3% in women rank 12 out of 82 ICD-10 MSD-diagnosis groups)), recovery is also considered if the person was free of MSD for at least 1 year. Table A2 provides an overview of the prevalence proportions of the six MSD subgroups in the study population (s. Online Resource 1).

### Labour force status

Employers, the Statutory Pension Fund and the Federal Employment agency are obliged to report on the labour force status and other socioeconomic characteristics (e.g. education, income) of each individual to the statutory health insurance provider. The reported labour force status provides information on whether a person is in active labour, receives unemployment benefits, is retired due to old age or disability, is in education, or is economically inactive. Therefore, the data contain employment histories showing periods of employment, unemployment, pension and other economic inactive episodes.

In line with the official European statistics^25^, we followed the labour force concept of the International Labour Organization (ILO), and defined Working Life Expectancy (WLE) as years spent in the labour force (i.e. in periods in employment and unemployment). To calculate WLE, individual insurance episodes spent in active labour were added to episodes in which unemployment benefits have been received. Years spent outside the labour force were based on the episodes in retirement due to old age or disability, in education, or in another economically inactive state (for a fully comprehensive description of the data and the definition of the labour force, see^4^). Accordingly, Healthy Working Life Expectancy depicts years in the labour force free of MSD.

### Educational inequalities

In this study, information on school-leaving qualification is used to analyse inequalities in MSD-free LE and MSD-free WLE. This information is coded within the occupational classification key^26^, which employers reported as part of their legal obligation. The data is therefore available for insured persons who ever had a paid job during the years the data are available for, i.e. 2005–2018. Educational level was classified in three groups: graduation after 8 to 11 years of schooling (*Volksschule, Hauptschule, Realschule* or equivalent, lower), after 12 to 13 years of schooling (*Abitur, Fachabitur* or equivalent, higher) and no school-leaving qualification or unknown qualification. Inequalities in MSD-free WLE and MSD-free LE were analysed for the group with lower and higher school education. The share of no school-leaving qualification or missing information is 30% (Table 1).

**Table 1:** Descriptive statistic of the study population: Number of individuals, labour force proportion, and incidence rate of musculoskeletal diseases (MSD) by period, education, and age.

	Number of individuals				Labour Force proportion		MSD incidence rate	
	Men		Women		Men	Women	Men	Women
	N	col%	N	col%	row%	row%	per 1.000 person-year^b^	
Period								
2006–2008	833,071	28.9	822,252	30.2	73.1	52.0	123	144
2011–2013	939,550	32.6	879,016	32.3	76.4	58.0	125	143
2016–2018	1,108,435	38.5	1,018,742	37.5	78.8	65.9	123	142
Education^a^								
Low	560,646	59.7	499,571	56.8	84.1	64.3	133	149
High	93,000	9.9	110,693	12.6	69.6	65.3	83	103
No certificate or unknown	285,904	30.4	268,752	30.6	60.2	41.0	123	155
Age								
18–29	777,597	27.0	695,235	25.6	72.9	63.4	76	85
30–39	532,131	18.5	477,362	17.6	90.7	69.5	113	127
40–49	608,191	21.1	568,754	20.9	89.5	69.2	142	169
50–59	568,432	19.7	549,442	20.2	83.3	65.5	163	198
60–69	394,705	13.7	429,218	15.8	39.1	24.4	160	191

^a^Values refer to the second period only; ^b^Under risk: in case of incidence only MSD-free person-years.

Data source: AOK Lower Saxony health insurance data.

### Statistical analyses

Healthy Life Expectancy defined as the number of expected years spent free of MSD is calculated using multistate life tables. Three states were considered: healthy (free of MSD), unhealthy (MSD prevalent) and dead. The calculation of the life years spent in the labour force free of MSD (MSD-free WLE) is calculated based on a five-state multistate life table: (1) healthy non-labour force, (2) unhealthy non-labour force, (3) unhealthy labour force, (4) healthy labour force, and (5) death. MSD-free LE and MSD-free WLE were calculated based on the age-specific transition rates between the five states.

Time trends in MSD-free LE and MSD-free WLE were analysed by comparing three time periods (2006–2008, 2011–2013, and 2016–2018). Educational inequalities are reported for the middle period 2011–2013 only, as missing values were unevenly distributed over time (for further information, see^4^). To draw a more differentiated picture of time trends and inequalities, all expectancies are reported at the beginning of the working biography (age 18) and at higher working age (age 50 and 60). Since the current legal retirement age in 2024 in Germany is age 66 (birth cohort 1958), and only a very small percentage works beyond the age of 69, the upper age limit for all analyses is set to age 69. Therefore, all expectancies are reported as partial life expectancies up to the age of 69.

Detailed information on time trends and educational inequalities in total working life expectancy (healthy plus unhealthy life years) and an in-depth discussion of methodological and data-related issues can be found in a previous publication using exactly the same study population and periods^4^. In short, WLE increased strongly across the study period, especially among women. Furthermore, educational inequalities in total WLE were most pronounced at older ages with people with higher education having higher WLE than their lower-educated counterparts.

### Ethics declarations and consent to participate

The analyses of this study are based on a pre-existing claims dataset collected as part of the routine administrative activities of a statutory health insurance provider. The use of this kind of data is regulated by German law (German Civil Code “Bürgerliches Gesetzbuch”). The data protection officer of the Local Statutory Health Insurance of Lower Saxony-AOK Niedersachsen (Germany) has given permission to use the data for scientific purposes. Due to these regulations, neither an ethical approval nor consent to participate was required for this study. We confirm that all data were fully anonymized before we accessed them. We confirm that all methods were carried out in accordance with relevant guidelines and regulations.

## Results

### Time trends in MSD-free LE and MSD-free WLE

Descriptive statistics of the study population are shown in Table 1. Across all periods, the crude incidence rate of MSD increases with age, is higher among women than among men, and did not change significantly over time. Incidence is also higher among less educated than among higher-educated individuals. Furthermore, the labour force proportion is much higher in men than in women, with a difference of around 20%-age points in each period (Table 1).

**Figure 1:**
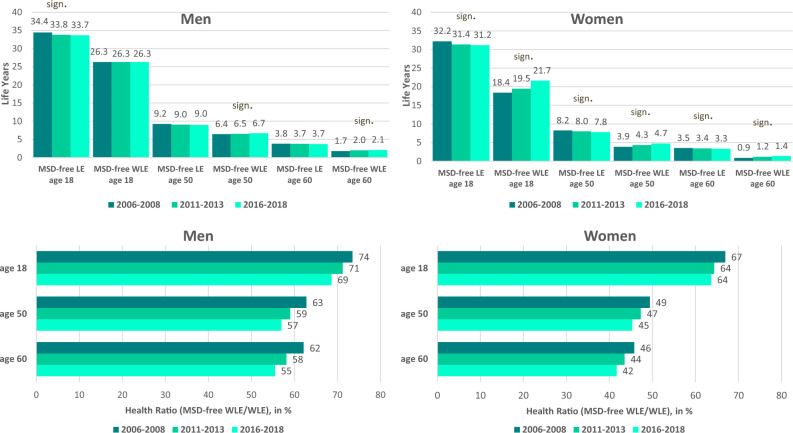
Time trends in musculoskeletal disease-free Life Expectancy (MSD-free LE) and musculoskeletal disease-free Working Life Expectancy (MSD-free WLE) and the proportion of MSD-free WLE in total WLE (Health Ratio) by period, gender and age (Note: MSD-free WLE and MSD-free LE are given as partial expectancies up to age 69; sign. significant increases in MSD-free LE or MSD-free WLE over time based on bootstrapped 95%-CIs, 95%-CIs are displayed in Online Resource 1, Table A1. Data source: AOK Lower Saxony health insurance data.

Over time, MSD-free life years (MSD-free LE) at age 18 decreased slightly in men (34.4 to 33.7 years) and women (32.2 to 31.2 years). At older working ages, MSD-free LE remained rather stable as changes over time were non-significant (Fig. 1). MSD-free WLE increased in women at younger (18.4 to 21.7 years at age 18) and higher working age (3.9 to 4.7 years at age 50), while in men only minor changes were found (Fig. 1). Similar to MSD-free LE, MSD-free WLE in women is generally lower than in men (Fig. 1).

The proportion of MSD-free WLE in WLE (Health Ratio) at age 18 decreased from 74 to 69% in men and from 67 to 64% in women. Decreases at higher working age were even stronger (62% to 55% in men and 46% to 42% in women) (Fig. 1).

### Educational inequalities in MSD-free LE and MSD-free WLE

**Figure 2:**
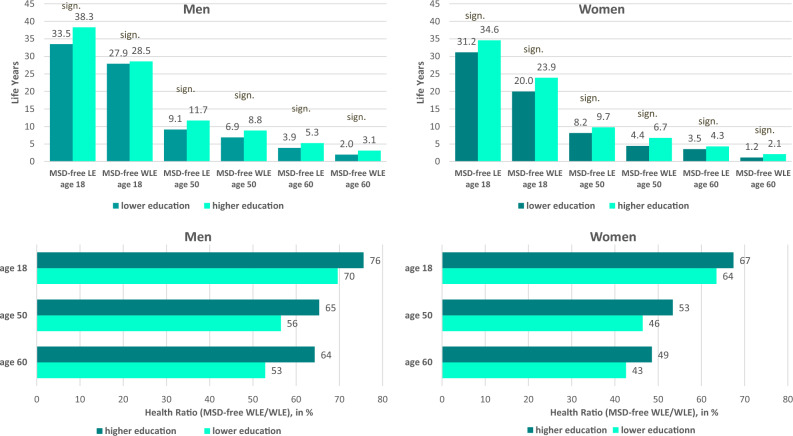
Educational inequalities in musculoskeletal disease-free Life Expectancy (MSD-free LE) and musculoskeletal disease-free Working Life Expectancy (MSD-free WLE) and the proportion of MSD-free WLE in total WLE (Health Ratio) by educational level, gender and age (Note: MSD-free WLE and MSD-free LE are given as partial expectancies up to age 69;sign. significant differences in MSD-free LE or MSD-free WLE between educational groups based on bootstrapped 95%-CIs, 95%-CIs are displayed in Online Resource 1, Table A1. Data source: AOK Lower Saxony health insurance data).

The Healthy Life Expectancy in terms of MSD differs clearly by educational level: People with a lower level of education can expect significantly fewer MSD-free life years than people with a higher level of education. While women at age 18 with higher educational attainment can expect to spend 3.4 more healthy years (34.6 vs. 31.2 years) up to age 69, the difference is even higher among men (Δ4.8 years, 38.3 vs. 33.5 years). A similar pattern was also found at higher working age (Fig. 2). Taking into account the inequalities in incidence and total WLE, educational inequalities were also found in terms of the number of years spent in the labour force free of MSD (MSD-free WLE). For men, they become most apparent at older working age (Δ2.1 years at age 50) and for women at younger (Δ4.1 years at age 18) and higher working age (Δ2.2 years at age 50) (Fig. 2).

Furthermore, people with a high school-leaving qualification can expect to spend more of their years in the labour force free of MSD (Health Ratio). This holds for both genders (e.g. 76% vs. 70% in men and 68% vs. 63% in women at age 18). Among men, the difference widened with age (Δ7 percentage points at age 18, Δ12 percentage points at age 60).

## Discussion

### Main findings

In this paper, we investigated whether the current trend towards longer working lives leads to an increase in disease-free years or whether these additional years in the labour market are accompanied by musculoskeletal diseases. To our knowledge, this is the very first study to investigate time trends in HWLE based on the absence of MSD, and the first one in which social inequalities in musculoskeletal disease-free WLE have been analysed. Healthy life years between age 18 and 69 decreased slightly during the study period, even though life expectancy increased over time^27^. This resulted in an increase in the length of life spent with MSD. Due to the very strong increase in WLE among women in Germany^4^, the absolute number of years in the labour force without MSD increased in women since 2006–2008 despite the general trend of decreasing MSD-free life years in the population. Since the increase in WLE in men was weaker than in women^4^, working life years without MSD remained nearly the same in men, which indicates that the additional years in the labour market are spent in the presence of MSD. However, the increase in WLE clearly exceeded the increase in MSD-free WLE. The negative health trend becomes most apparent in the marked decline in the proportion of years spent in the labor market that are free of MSD, which was found in both genders and in all age groups considered.

Furthermore, substantial educational inequalities in MSD-free LE and MSD-free WLE were found. As a result, men and women with lower education can expect less healthy life years in general and less healthy working life years in terms of MSD. Again, this becomes most apparent when the proportion of MSD-free WLE on total WLE is considered, as people with higher education spent a much greater share of their remaining time in the labour market free of MSD. Due to the higher incidence, the general level of the proportion of MSD-free WLE is lower in women than in men. However, the largest inequalities in the proportion were found in older men.

### Main findings in the light of previous research

So far, there are no studies on the development of HWLE over time in terms of working life years free of MSD. Therefore, our results cannot be directly compared with previous research. Up to now, there is only one study on trends in working life years spent with specific chronic diseases. This study reports increases in working life years spent with arthritis and rheumatism^16^, which is in line with our results since the share of MDS-free life years in total working life years decreased in our study despite the general increase in WLE^4^. Based on other health indicators like long-standing illnesses, self-rated health or disabilities, most studies reported increasing HWLE over time which, however, was accompanied by increases of years spent in the labour market and in poor health^3,14, 15^. With respect to MSD, time trends are even worse since MSD-free WLE only increased in women and MSD-free life years in the general working-age population tended to decrease over time.

Overall, the evidence on time trends in MSD is scarce. Previous studies usually did not focus on disease prevalence or incidence but on disabilities or pain due musculoskeletal disorders. However, while the prevalence of pain mostly showed inconsistent time trends, most studies on trends on disability due to MSD reported increases over time^28^. Furthermore, social inequalities in musculoskeletal disorders have been previously reported^29,30^ supporting our results since this also fosters disparities in H(W)LE between groups with different education. For Germany, most of the available research on trends in MSD is based on health insurance data. Our findings on the decline in MSD-free LE are supported by the rising age-standardised rates reported^31^ along with increasing MSD-related drugs prescriptions^32^ and increasing numbers of sick-leave days due to MSD^33^ for the time period 2006 to 2018.

### Strengths and limitations

There are several advantages of using health insurance data for our study: The data combines detailed information on diagnosis codes of specific diseases, mortality, socio-economic status, and employment histories in a single data set. This made it possible to perform analyses on time trends and social inequalities in WLE free of specific diseases, which has not been performed so far in a similar way. Using this data, multistate life table analyses based on age-specific transition rates could be performed, which is considered the preferred method for determining health or working expectancies^34^. So far, research on WLE and HWLE was based mostly on more general health indicators (such as disability or SRH). Broadening research to specific diseases is particularly important because it depicts the disease burden in working lives and thus enables specific prevention needs to be identified. The use of health insurance data can therefore make a valuable contribution to enhance the knowledge about trends and inequalities in healthy working lives.

One limitation of the data relates to the availability of educational information. Since educational attainment is only available for those who were ever in paid employment between 2005 and 2018, it is often missing for long-term unemployed and non-working individuals. Furthermore, a more differentiated classification of education may have enabled a deeper understanding of the educational inequalities in HWLE but could not be applied due to data restrictions. However, as discussed in a previous paper^4^, the educational inequalities in WLE are comparable to earlier studies^1–3^. Consistent with previous research on inequalities in musculoskeletal disorders^10,29, 30^, the educational differences found for MSD-free (working) life years also point in the expected direction.

Since we use routinely collected data, the analyses are unaffected by health-related non-response, which may lead to an overestimation of good health in survey data. However, while age and sex distributions are comparable to the general population, individuals with high SES are underrepresented^35^. This affects the level of WLE and MSD incidence. However, the focus of this paper is on time trends of MSD-free (working) life years which we assume are unaffected. While incidence may be somewhat higher than in the total population, previous findings on trends in prevalence^31^, prescribed drugs^32^ and sick-leave days due to musculoskeletal disorders^33^ in Germany are supporting our results on increasing disease rates over time.

WLE can either be defined as years spent in the labour force or in actual employment (e.g.^2,36^). In this paper, WLE is based on the ILO labour force concept, which means that episodes of employment and unemployment contribute to WLE. One advantage of this approach is that WLE depicts the potential of years of employment under favourable labour market conditions. This approach is useful if the limits and possibilities of extending the length of working life are discussed from a health perspective alone, i.e. ignoring economic framework conditions, which may change over time. Furthermore, this approach allowed us to compare trends in WLE to previous studies or Eurostat^1,3, 25, 36^ and to assess whether the time trends in the insurance population are comparable to the trends in the general population. However, although studies reporting WLE based on actual employment are more sensitive to changing economic conditions, they also clearly contribute to the current state of research and it depends on the research question which approach is to be preferred.

### Future perspectives and implications of our findings

From a public health perspective, studies focusing on MSD are important because of the high prevalence of MSD, the variety and severity of the associated complaints, and their association with early labour market exits. The decrease in healthy life years illustrates that MSD is a serious public health concern that already places a heavy burden on both the general population and the labour force. Previous research shows that MSD are associated with adverse health behaviors, such as the lack of physical activity and obesity^28^. With respect to MSD, it therefore seems challenging if the working life is to be extended beyond the current pension age. In particular, this applies to groups that already have a high prevalence of MSD and working conditions with high levels of physical workload. In Germany, there is a clear disparity in the number of sick-leave days between occupational groups, which suggest that maintaining the workability up to higher ages may be challenging^29^. Especially for these groups, it appears to be a major public-health issue to counteract or deal with the increasing health burden due to musculoskeletal disorders. The promotion of job-specific preventive approaches available in Germany could be helpful, such as the employer’s obligation for risk assessment including physical workload or the offer of occupational preventive medical consultations and return-to-work programs for employees.

## Conclusions

The study clearly shows that the extension of working life is not accompanied by a comparable increase in the number of years spent without musculoskeletal diseases in the labour force. Instead, the proportion of healthy years in total WLE is decreasing, and increased years in the labour market are associated with musculoskeletal disorders. This development shows that musculoskeletal diseases are a serious public health concern and effective prevention strategies are urgently needed to maintain the workability of the labour force population. Previous prevention strategies have mostly targeted occupations with high physical exposures, which are most common in the blue-collar sector, where traditionally more men than women work in Germany. Our analyses show that women also spend many working years with musculoskeletal diseases and should be a greater focus of future prevention efforts. Due to the large disparity between educational groups, special attention should also be paid to individuals with lower education.

Further research should focus on inequalities in disease-free life years between individuals with different occupations and occupational exposures in order to tackle the question of the effect of work-related inequalities on healthy life years in more depth.

### Supplementary Information

Supplementary Tables.

## Data Availability

The data analysed in this study cannot be made publicly available due to protection of data privacy of the insured individuals by the AOK Niedersachsen (AOKN-Statutory Local Health Insurance of Lower Saxony). The data underlying this study belong to the AOKN. Researchers interested in the data supporting the conclusions of this article can send data access requests to the AOK Niedersachsen using the following e-mail address: AOK.Service@nds.aok.de.

## Abbreviations

MSD: Musculoskeletal diseases
WLE: Working life expectancy
HWLE: Healthy working life expectancy
HLE: Healthy life expectancy
HRQoL: Health-related quality of life
MSD-free LE: Musculoskeletal diseases-free life expectancy
MSD-free WLE: Musculoskeletal diseases-free working life expectancy
ILO: International Labour Force Organization
