# Supplementary material for: The development of working life expectancy without musculoskeletal diseases against the backdrop of extended working lives
Source: Sci Rep. 2024 Apr 4;14:7930. doi: 10.1038/s41598-024-58650-2 (PMC10994922; doi:10.1038/s41598-024-58650-2)
Supplement: Supplementary file 1 — Supplementary Tables. [file 41598_2024_58650_MOESM1_ESM.pdf]

Scientific Reports

The development of working life expectancy without musculoskeletal diseases against the backdrop of extended working lives

Juliane Tetzlaff[1*], Jelena Epping[1], Jona Theodor Stahmeyer[2], Falk Liebers[3], Janice Hegewald[3], Stefanie Sperlich[1], Johannes Beller[1]; Fabian Tetzlaff[4]

[1] Medical Sociology Unit, Hannover Medical School, Hanover, Germany
[2] AOK Niedersachsen- Statutory Health Insurance of Lower Saxony, Hannover, Germany
[3] Federal Institute for Occupational Safety and Health (BAuA), Berlin, Germany

[4] Division of Social Determinants of Health, Department of Epidemiology and Health Monitoring, Robert Koch-Institute, Berlin, Germany

*****Corresponding author

Medical Sociology Unit, Hannover Medical School, Carl-Neuberg Str. 1, 30625 Hanover, Germany

E-mail: Tetzlaff.Juliane@mh-hannover.de

Table A1 Musculoskeletal disease-free Life expectancy (MSD-free LE) and musculoskeletal disease-free Working Life Expectancy (MSD-free WLE) by period, education, gender and age 95%-Confidence Intervals[1)]

		Men					
		MSD-free LE			MSD-free WLE		
**Age**		**18**	**50**	**60**	**18**	**50**	**60**
**Period**	**2006-2008**	34.4 (34.4-34.5)	9.2 (9.2-9.3)	3.8 (3.8-3.8)	26.3 (26.2-26.4)	6.4 (6.4-6.5)	1.7 (1.7-1.8)
	**2011-2013**	33.8 (33.8-33.9)	9.0 (9.0-9.1)	3.7 (3.7-3.8)	26.3 (26.2-26.3)	6.5 (6.5-6.5)	2.0 (1.9-2.0)
	**2016-2018**	33.7 (33.6-33.7)	9.0 (8.9-9.0)	3.7 (3.7-3.7)	26.3 (26.2-26.4)	6.7 (6.6-6.7)	2.1 (2.1-2.1)
**Education[2)]**	**low**	33.5 (33.4-33.6)	9.1 (9.1-9.2)	3.9 (3.8-3.9)	27.9 (27.8-28.0)	6.9 (6.8-6.9)	2.0 (1.9-2.0)
	**high**	38.3 (38.0-38.6)	11.7 (11.5-11.9)	5.3 (5.0-5.5)	28.5 (28.3-28.8)	8.8 (8.7-9.1)	3.1 (2.9-3.3)

		Women					
		MSD-free LE			MSD-free WLE		
**Age**		**18**	**50**	**60**	**18**	**50**	**60**
**Period**	**2006-2008**	32.2 (32.1-32.3)	8.2 (8.2-8.3)	3.5 (3.5-3.5)	18.4 (18.3-18.4)	3.9 (3.8-3.9)	0.9 (0.9-0.9)
	**2011-2013**	31.4 (31.3-31.4)	8.0 (8.0-8.1)	3.4 (3.4-3.4)	19.5 (19.4-19.6)	4.3 (4.3-4.4)	1.2 (1.2-1.2)
	**2016-2018**	31.2 (31.1-31.2)	7.8 (7.8-7.8)	3.3 (3.3-3.3)	21.7 (21.6-21.7)	4.7 (4.7-4.8)	1.4 (1.4-1.4)
**Education[2)]**	**low**	31.2 (31.1-31.2)	8.2 (8.1-8.2)	3.5 (3.5-3.6)	20.0 (19.8-20.0)	4.4 (4.4-4.5)	1.2 (1.2-1.2)
	**high**	34.6 (34.3-34.9)	9.7 (9.5-10.0)	4.3 (4.1-4.6)	23.9 (23.8-24.3)	6.7 (6.5-7.0)	2.1 (2.0-2.3)

1)Confidence intervals are bootstrapped using 1000 replications

2)values refer to educational inequalities in 2011-2013

MSD-free LE and MSD-free WLE are given as partial expectancies up to age 69

Table A2 Descriptive statistics on the prevalence proportion[1)] of musculoskeletal diseases (MSD) in study population aged 18-69 years by MSD subgroup (in %)

		Men	Women
**MSD subgroup**	**Arthropathies (M00-M25)**	11.36	15.15
	**Systemic connective tissue disorders (M30-M36)**	0.37	0.95
	**Dorsopathies (M40-M54)**	20.32	25.7
	**Soft tissue disorders (M60-M79)**	7.07	10.78
	**Osteopathies and chondropathies (M80-94)**	1.74	3.57
	**Other disorders of the musculoskeletal system and connective tissue (M95-M99)**	2.49	3.77

Note: [1)]Combined for in the three analysed periods (2006-2008, 2011-2013, 2016-18); MSD subgroups are classified according to ICD-10 (codes shown in brackets), Data source AOK Lower Saxony
